# Detection of human papillomavirus (HPV)-specific T cell response in women with low-grade cervical intraepithelial lesion and HPV vaccinated subjects

**DOI:** 10.3389/fimmu.2025.1733404

**Published:** 2026-02-12

**Authors:** Paola Zelini, Federica Zavaglio, Mattia Dominoni, Marta Valsecchi, Lucrezia Lo Grasso, Piera d’Angelo, Federica Giardina, Greta Romano, Stefania Paolucci, Daniele Lilleri, Irene Cassaniti, Arsenio Spinillo, Fausto Baldanti, Barbara Gardella

**Affiliations:** 1 Microbiology and Virology Department, Fondazione IRCCS Policlinico San Matteo, Pavia, Italy; 2 Department of Clinical, Surgical, Diagnostics and Pediatric Sciences, University of Pavia, Pavia, Italy; 3 Department of Obstetrics and Gynecology, Fondazione IRCCS Policlinico San Matteo, Pavia, Italy

**Keywords:** human papillomavirus, vaccination, cervical lesion, genotype, cellular immunity

## Abstract

**Background:**

Human papillomavirus (HPV) is the most common sexually transmitted infection worldwide and HPV-driven cervical cancers remain a major health concern. This study aimed primarily to develop a test for assessing and characterizing HPV-specific T-cell responses, in HPV-vaccinated women and women with cervical intraepithelial neoplasia (CIN)1.

**Methods:**

T-cell responses against HPV-16 and 18 L1, E6, and E7 proteins were evaluated by flow cytometry with a 24h activation-induced marker (AIM) and a 7-day lymphoproliferation (LPR) assays in 18 vaccinated and 60 CIN1 women. HPV genotyping was performed on vaginal swab samples.

**Results:**

LPR assay demonstrated higher sensitivity than AIM. T-cell response was mainly directed against L1 and was higher in CD4+ than CD8+ T cells. All vaccinated women exhibited CD4+ T-cell responses against HPV-16 and to a lesser extent HPV-18 L1. Among CIN1 patients, 46.6% and 33.3% showed HPV-16 and -18 L1-specific CD4+ responses, respectively, even when infected with other HPV strains. Responses were predominantly associated with TH1 and TH17 phenotypes.

**Conclusions:**

The LPR assay is a sensitive tool for detecting HPV-specific T-cell responses. The presence of T-cell responses in CIN1 patients infected with non-16/18 HPVs suggests potential cross-reactivity among HPV genotypes.

## 1 Introduction

Human papillomavirus (HPV) is the most common sexually transmitted infection (STI) in the world (1) since the risk of being infected at least once in a lifetime among both men and women is nearly 50% (2). HPV is a small, double-stranded circular DNA virus, with more than 228 genotypes classified into five genera (alpha, beta, gamma, mu, and nu) (3). The alpha genus HPV types infect both cutaneous and mucosal epithelia and are classified into low-risk (LR) and high-risk (HR) genotypes, based on their potential to cause cancer. HR genotypes, including HPV 16, 18, 31, and 45, are responsible for the majority of HPV-related cancers, such as cervical, head and neck, penile, anal, vaginal, and vulvar cancers (4). In contrast, LR strains (e.g., HPV 6 and 11) are mainly associated with warts on the genitals, anus, mouth, and throat (5). HPV is associated with 4.5% of all cancer and 8.6% of cancers in women, including cervical cancer (CC), which is the fourth most common cancer in terms of incidence and mortality in women (6).

Persistent infection with high-risk strains of the HPV is the cause of 99.7% of cervical cancer cases (7). So far, the risk of occurrence also depends on regional conditions and geographic variability of viral variants (1). It has been reported that of the 448 types documented, only 12 are currently classified as carcinogenic, including 16, 18, 31, 33, and 52 and types (8). Focusing on HPV-related cervical lesions, a persistent HPV infection can be established, increasing the risk of progression from low grade cervical intraepithelial neoplasia (CIN1) to middle/high grade (CIN2/3) or invasive cancer. It is a matter of fact that HR-HPV infection is the main factor associated with development of cervical cancer. However, only 1% of women infected with HR-HPV will gradually develop cervical cancer (9, 10), and after HR-HPV infection, the large majority of the patients spontaneously clears the infection because of their immune responses. Thus, immune response, especially T cell immunity, seems to be a key factor in prognosis of HPV infection and its malignancy (11–14). Consequently, the role of immune response seems to be crucial in balancing between persistence and clearance of infection. The introduction of prophylactic vaccines in early childhood has reduced the HPV-related tumor incidence (15).

HPV uses as variety of immune evasion mechanisms to suppress immune responses and promote cancer progression (16), including the limited viral antigen presentation to host immune system during natural infection. Of note, HPV infections are poorly immunogenic, since only 40 to 60% of the women with positive HPV DNA at the cervix seroconversion (8). All these aspects complicate the study of immune response against HPV, in particular of the T-cell response at peripheral level. A recent study demonstrated that the low or undetectable levels of HPV E1-specific T-cell response was associated with poor prognosis in patients with cutaneous squamous cell carcinoma (17). Moreover, recent studies suggest that E7-specific T cell response detected in peripheral blood seems to be associated with HPV- 16 clearance in infected patients (18).

Here, we aim to implement and assess an immunological assay for the quantification and phenotypical characterization of HPV-specific T cells in a group of women with CIN1 at time of diagnosis. As control, a group of vaccinated healthy women was included. Overall, in this preliminary study we evaluated HPV genotypes and specific T-cell response to L1, E6, and E7 proteins in a cohort of women with low grade CIN (CIN1). The future aim will be to correlate the T-cell response with lesion progression or regression, in order to define risk stratification for lesion progression, taking into account that the majority of lesions undergo spontaneous regression (60–80% clearance within two years) (19).

## 2 Materials and methods

### 2.1 Study design

HPV-specific T-cell immunity was measured in a group of 18 healthy women (median age 29 years, range 26-32) vaccinated with two or three dose of Gardasil 9 (median time since the first dose 6 years, range 2-14). Furthermore, HPV-specific T-cell response was measured in 60 CIN1 patients (median age 43 years, range 27-68) with who did not receive HPV vaccine. Since this was an exploratory study, no power calculation was done for sample size determination. Enrollment was conducted between March 2023 and November 2023 at time of diagnosis at the Obstetrics and Gynecology Clinics of Fondazione IRCCS Policlinico San Matteo and diagnosis was based on cytological analysis of pap- smear. Women with low-grade abnormal Pap smears were enrolled according to the cytological classification applied during the study period. The patients were referred by the cytological screening service, other institutions, and private practitioners. Exclusion criteria were continuing pregnancy, past HPV infection or therapy for CIN or total hysterectomy before enrollment, a biopsy indicating CIN2+, and HPV immunization prior to participation. Two distinct gynecologists (BG and MD) certified by the Italian Society of Colposcopy conducted a standardized colposcopic examination. The colposcopic examination followed international colposcopy nomenclature (12). Targeted cervical biopsies were performed regardless of the colposcopic impression. When the extent of the lesion or the squamocolumnar junction was not completely apparent (NTZ Type 3), endocervical curettage was performed as determined by the clinician. Histological diagnosis were made by two qualified gynecological pathologists who reached a consensus of our department. The clinical and demographic characteristics of CIN1 patients are shown in **Table 1**. The patients data reported in the **Table 1** were collected at the time of enrollment using a form specifically prepared for this study. After diagnosis, heparinized peripheral blood samples, and vaginal swabs were collected for characterization of peripheral blood T-cell response and HPV genotyping. The study procedures were approved by the local Ethics Committee (P-08068322) and women participating in the study gave written informed consent.

**Table 1:** Characteristics of the 60 enrolled patients with CIN1 lesion.

Age, yrs (median, IQR)	47 (27-65)
Ethnicity, n. (%)	
Caucasian	60 (100.0)
Menopausal status, n. (%)	
Yes	26 (43.3)
No	34 (56.6)
Comorbidity, n. (%)	
Yes	37 (61.6)
No	23 (38.3)
Smoking, n. (%)	
Yes	21(35.0)
No	29(48.3)
Unknown	10 (16.7)
Contraceptive use, n. (%)	
Yes	44 (73.3)
No	16 (26.6)
Cervical cytology, n. (%)	
ASCUS	8 (13.3)
L-SIL	52 (86.6)
HPV genotype, n. (%)	
84	2 (3.3)
61	1 (1.6)
62	1 (1.6)
82	1 (1.6)
53	6 (10.0)
56	1 (1.6)
66	9 (15.0)
18	1 (1.6)
59	1 (1.6)
70	2 (3.3)
45	1 (1.6)
16	5 (8.3)
58	6 (10.0)
31	7 (11.6)
33	1 (1.6)
52	1 (1.6)
6	1 (1.6)
11	1 (1.6)
73	1 (1.6)
120	1 (1.6)
undefined	10 (16.6)

### 2.2 HPV genotyping

Viral DNA was extracted from 400µl of cervical swabs using QIAsymphony DSP Virus/Pathogen Midi kit (Qiagen, Heidelberg, Germany) on QIAsymphony platform (Qiagen, Heidelberg, Germany), with a final elution in 60µl. HPV genotyping was per-formed by amplifying a partial region of L1 gene using primers reported by Gravitt et al, 1998 (20). Briefly, the reaction mix was prepared using AmpliTaq Gold™ DNA Polymerase (Life Technologies, NJ, USA), according to manufacturer’s instruction. Thermal profile was as follows: 10 min at 95 °C, 50 cycles of 1 min at 94 °C, 1 min at 55 °C and 1 min at 72 °C. Final hold was 10min at 72 °C. The proper amplicons obtained (size expected ~450 bp) were sequenced with BigDye™ Terminator v1.1 Sequencing kit (Life Technologies, NJ, USA) on 3500xL Dx Genetic Analyzer (Applied Biosystems, NJ, USA). The resulting sequences were analyzed using Sequencher software, version 5.0 (Gene Codes Corporation, Ann Arbor, MI, USA). BLAST analysis (https://blast.ncbi.nlm.nih.gov/Blast.cgi) was performed to identify the HPV genotype.

### 2.3 Protein peptide pools

Peptides pools (15 amino acids -aa- in length with an 11 amino acid overlap) spanning the entire L1, E6, and E7 proteins of HPV-16 (124 peptides for L1 -length 505 aa- for L1; 37 peptides for E6 - length 158 aa-; 22 peptides for E7 -length 98 aa-) and of HPV-18 (140 peptides for L1 -length 568 aa-; 37 peptides for E6 -length 158 aa-; 24 peptides for E7 -length 105 aa-) were used (JPT, Peptide Technologies, Berlin, Germany). A peptide pool of human actin (15 mers, overlapping by 10 amino acids, Pepscan, Lelystad, The Netherlands) was used as negative control.

### 2.4 Activation induced markers assay

To evaluate antigen-specific T cell activation, peripheral blood mononuclear cells (PBMCs) were stimulated for 20 h with the above mentioned HPV and control peptide pools [1 µg/mL] in the presence of co-stimulator antibodies CD28 and CD49d (BD Bio-sciences). Cell were seeded in 96-wells round bottom plates at a density of 0.5–1 × 106 cells/200 µL culture medium for well. Culture medium was RPMI 1640 (Euroclone) supplemented with 2 mM L-glutamine (Euroclone), 100 U/mL penicillin and 100 µg/mL streptomycin solution (Euroclone), and 10% of heat-inactivated FBS.

After culture, cells were washed with PBS 2 mM EDTA and stained in PBS with Live/Dead Fixable Violet Dye (Invitrogen) for 30 min at 4°C. After rising with PBS and staining in PBS 5% FCS with CD4 APC Cy7 (BD Biosciences), CD8 V500 (BD Biosciences), CD25 PECy7 (BD Biosciences) and CD137 PECy5 (BD Biosciences) antibodies for 30 min at 4°C, cells were washed and re-suspended in PBS 1% paraformaldehyde.

Antigen-specific T-cell frequency was determined by subtracting the frequency of activated (CD25^+^CD137^+^) CD4^+^ or CD8^+^ T cells detected in PBMC incubated with hu-man actin peptides from the frequency of activated CD4^+^ or CD8^+^ T cells detected in PBMC incubated with HPV peptides. A value < 0.05% antigen-specific T cells was considered negative while a value ≥ 0.05% was considered positive. Flow cytometry analyses were performed with a BD FACSLyric and BD FACSuite v1.5 software.

### 2.5 Lymphoproliferative response assay

PBMCs (600,000/200 μL culture medium for well) were stimulated in triplicate in 96-well round- bottom plates with peptide pools from HPV-16 and -18 and human ac-tin at a final concentration of 0.1 µg/mL for 7 days. Culture medium was RPMI 1640 (Euroclone, Milano, Italy) supplemented with 2 mM L-glutamine (Euroclone), 100 U/mL penicillin and 100 µg/mL streptomycin solution (Euroclone), 10% of heat inactivated FBS, 1 mM Sodium Pyruvate (Gibco, Grand Island, NY, USA), 100 µM non-essential amino acids (Euroclone), and 50 µM 2-Mercaptoethanol (Gibco). After culture, cells were washed, stained with Live/Dead Fixable Violet Dye (Invitrogen) and subsequently with CD3 PerCP 5.5 (BD), CD4 APC Cy7 (BD), CD8 FITC (BD), CD25 PECy7 (BD), CD278 (ICOS) APC (Invitrogen). Finally, cells were washed and resus-pended in PBS 1% paraformaldehyde. A Cell Proliferation Index (CPI) for anti-gen-specific expanded T-cells was determined by subtracting the percentage of CD25^+^ICOS^+^ CD3^+^CD4^+^ or CD3^+^CD8^+^ detected in PBMC incubated with actin pep-tides from the percentage of CD25^+^ICOS^+^ T-cell subsets detected in PBMC incubated with HPV peptides (21). A CPI <1.5% was considered negative while a value ≥ 1.5% was considered positive. Flow cytometry analyses were performed with a BD FACSLyric and BD FACSuite v1.5 software.

### 2.6 Data analysis

Quantitative data were showed as median and interquartile range (IQR) while qualitative data were showed as frequency or percentage. The non-parametric Mann-Whitney U-test was used for comparison of two unpaired groups while Wilcoxon test was used for comparison of two paired groups. Fishers’ test was used for qualitative data. Analyses were performed with Prism 8.3.0 Software (Graph Pad Software, San Diego, CA, USA). Flow cytometry analyses were performed using a FACS Canto II flow cytometer and analyzed with BD DIVA software.

## 3 Results

### 3.1 AIM and LPR assays for determination of HPV-specific T-cell response

**Figure 1:**
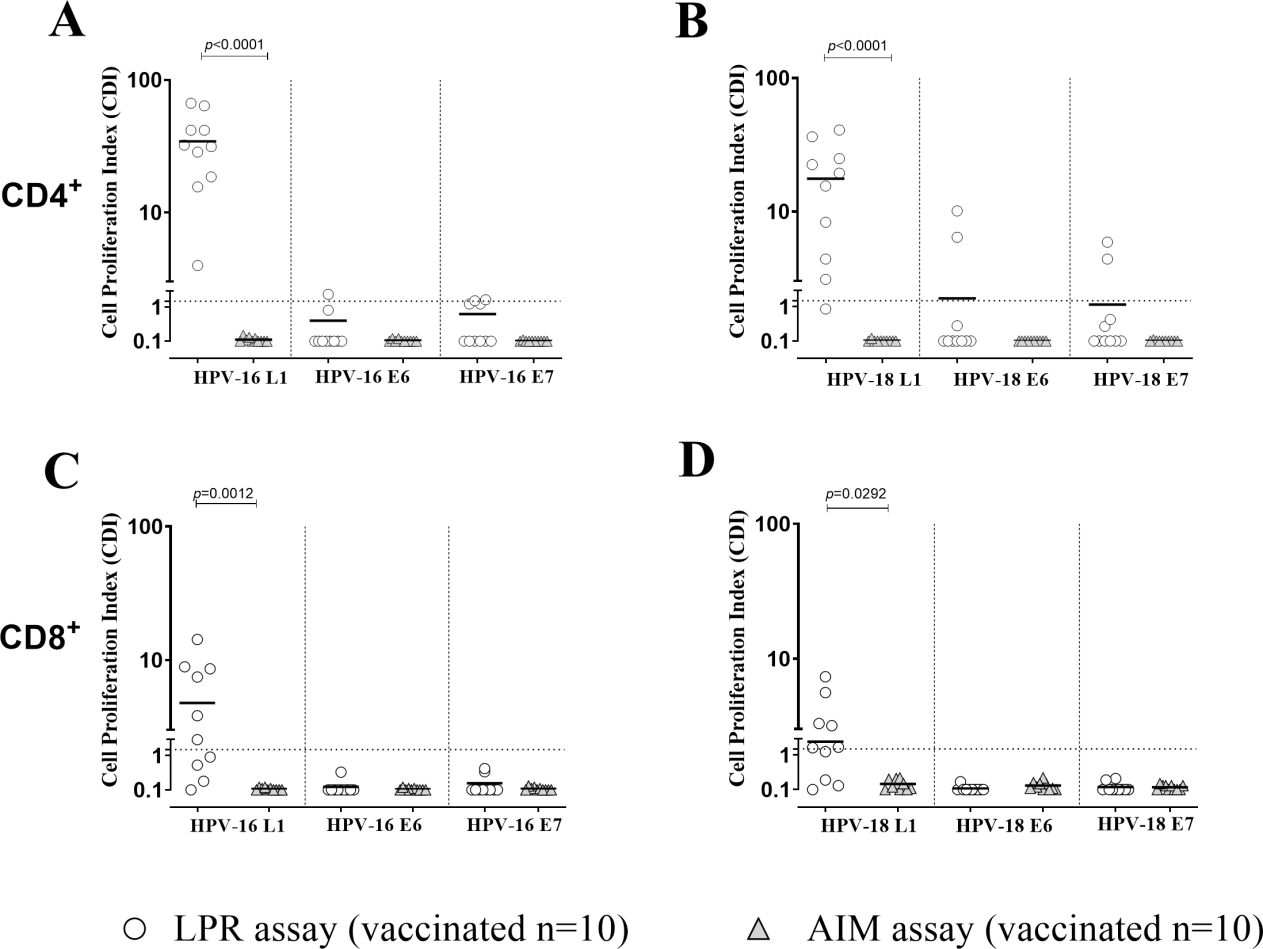
Cell proliferation index (CPI) for both CD4^+^**(A, B)** and CD8^+^ T cells **(C, D)** against HPV-16 and HPV-18 for antigens L1, E6 and E7 was measured using Lymphoproliferation (LPR; white dots) and Activation Induced Markers (AIM; grey triangles) assays in 18 HPV vaccinated healthy women. Significant p value were given for each graph. Dotted horizontal lines indicates cut-off of positive results.

HPV-16 and 18-specific T-cell response was evaluated in parallel by LPR and AIM assays in 10 vaccinated women (**Figure 1**). Overall, T-cell response was detected only when using LPR assay while response was undetectable when using AIM test. Looking at LPR assay, all vaccinated women developed a sustained CD4^+^ T-cell response against HPV-16 L1, while positive HPV-18 L1 response was observed in 9/10 (90%) women. Interestingly, median CD4^+^ T-cell response against HPV-16 L1 was significantly higher than that detected against HPV-18 L1 [31.9 IQR (17.8-47.3) vs 17.4 IQR (4.1-27.8) cell proliferation index (CPI); p=0.0020]. CD4^+^ T-cell response against HPV-16 E6 or E7 proteins was observed in 1/10 (10.0%) and 2/10 (20.0%) women, respectively. CD4^+^ T-cell response against HPV-18 E6 or E7 proteins was observed in 2/10 (20.0%) women for either antigen.

CD8^+^ T-cell response against HPV-16 and -18 L1 was detected only in 6/10 (60.0%) and in 7/10 (70.0%) vaccinated women, respectively. No positive CD8^+^ T-cell response against HPV-16 and -18 E6/E7 proteins was observed. A significantly higher CD4^+^ and CD8^+^ HPV-16 (p<0.0001 and p=0.0012) and - 18 (p<0.0001 and p=0.0292) T cell response against L1 with LPR assay was observed.

### 3.2 Evaluation of T-cell response against HPV-16 and -18 in vaccinated and naturally infected patients

**Figure 2:**
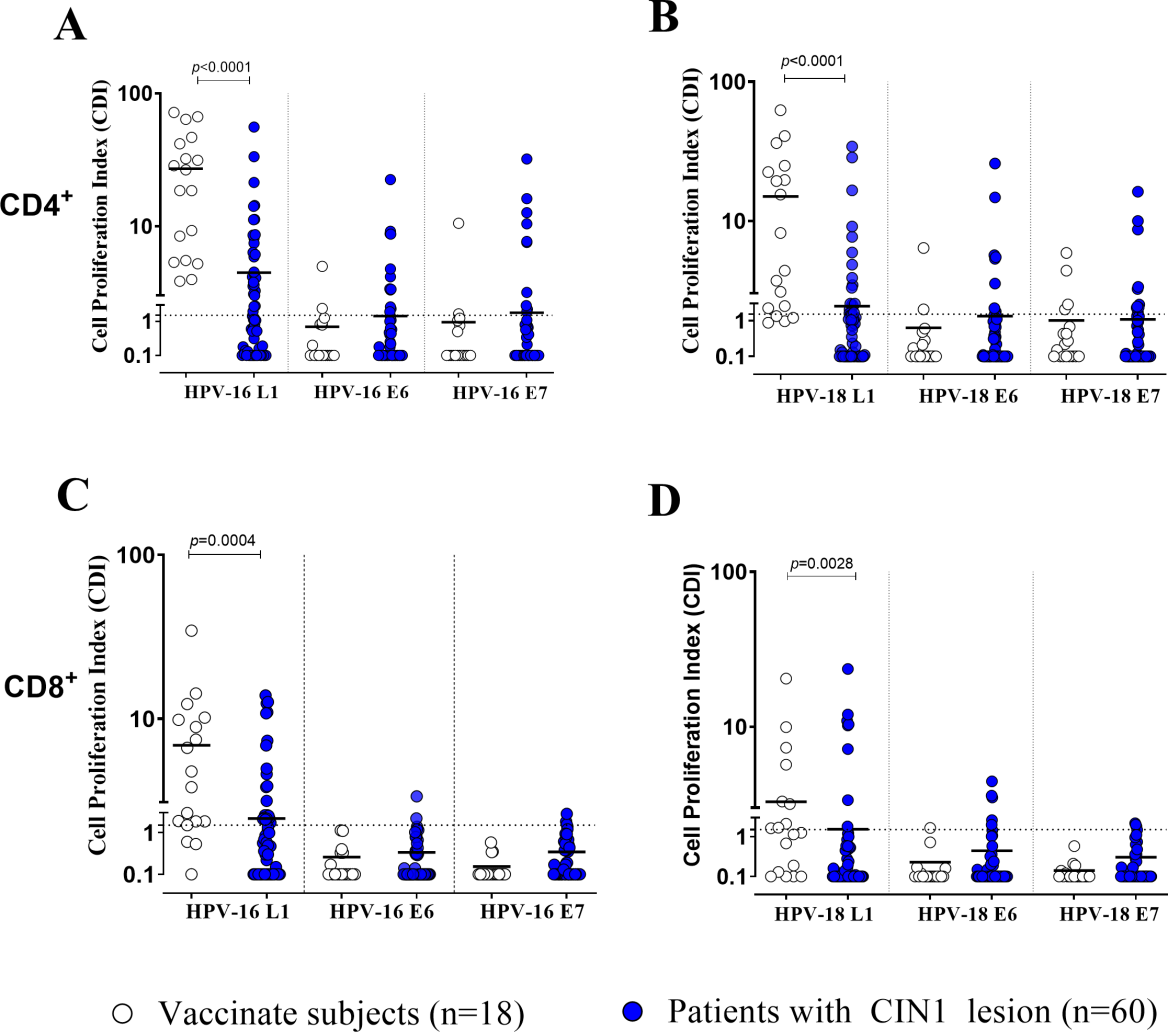
Cell proliferation index (CPI) for both CD4^+^**(A, B)** and CD8^+^ T cells **(C, D)** against HPV-16 and -18 for antigens L1, E6 and E7 was measured using Lymphoproliferation (LPR) in vaccinated women (white dots) and CIN1 patients (blue dots). Significant p value were given for each graph. Dotted horizontal lines indicates cut-off of positive results.

Based on preliminary results obtained in vaccinated healthy women, HPV-specific T-cell response in 60 CIN1 patients was evaluated using only LPR assay and results were compared with those obtained in 18 vaccinated women. While all the vaccinated women showed a sustained CD4^+^ T-cell response against HPV-16 L1, a positive response was observed in only 28/60 (46.6%) CIN1 patients (p<0.0001). On the other hand, positive CD4^+^ T-cell response against HPV-18 L1 antigen was observed in 14/18 (77.7%) vaccinated women and 20/60 (33.3%) CIN1 patients, respectively (p<0.0001) (**Figures 2A, B**). Looking at CD8^+^ T-cell response against HPV-16 L1 and HPV-18 L1, a positive CPI was observed in 15/18 (83.3%) vaccinated women vs 22/60 (36.6%) CIN1 patients (p=0.0009) and 11/18 (61.1%) vaccinated women vs 9/60 (15%) patients (p=0.0003), respectively (**Figures 2C, D**).

Notably, only 2/18 (11.1%) and 1/18 (5.5%) vaccinated women showed a positive CD4^+^ T-cell response against HPV-16 E6 and E7, while 2/18 (11.1%) and 4/18 (22.2%) against HPV-18 E6 and E7 antigens, probably related to a previous HPV infection. Only one vaccinated woman showed a CD8^+^ T cell response against HPV-18 E6.

Otherwise, a positive CD4^+^ T-cell response against HPV- 16 E6 or E7 was observed in 11/60 (18.3%) and 10/60 (16.6%) CIN1 patients and in 9/60 (15.0%) and 10/60 (16.6%) against HPV-18 E6/E7. A positive CD8^+^ T-cell response against HPV-16 E6 and E7 was observed in 2/60 (3.3%) and 2/60 (3.3%) CIN1 patients, respectively. For HPV-18 E6 and E7 was observed in 5/60 (8.3%) and 3/60 (5.0%) CIN1 patients.

CD4^+^ was higher than CD8^+^ L1-specific T-cell response for both HPV-16 and -18 (p<0.05). No difference between CD4^+^ and CD8^+^ T-cell response against E6 or E7 antigens was evaluated. In the **Supplementary Figure 1** (**Supplementary Figure S1**) the flow cytometry analyses and gating strategy was observed.

We conducted an analysis of the CIN1 patient group, stratified by category: menopausal and non-menopausal women, as these results must be interpreted considering the potential confounding effects of age and immune senescence. However, no statistically significant immunological differences were observed between the two groups, as showed in **Supplementary Figure 2** (**Supplementary Figure S2**).

**Figure 3:**
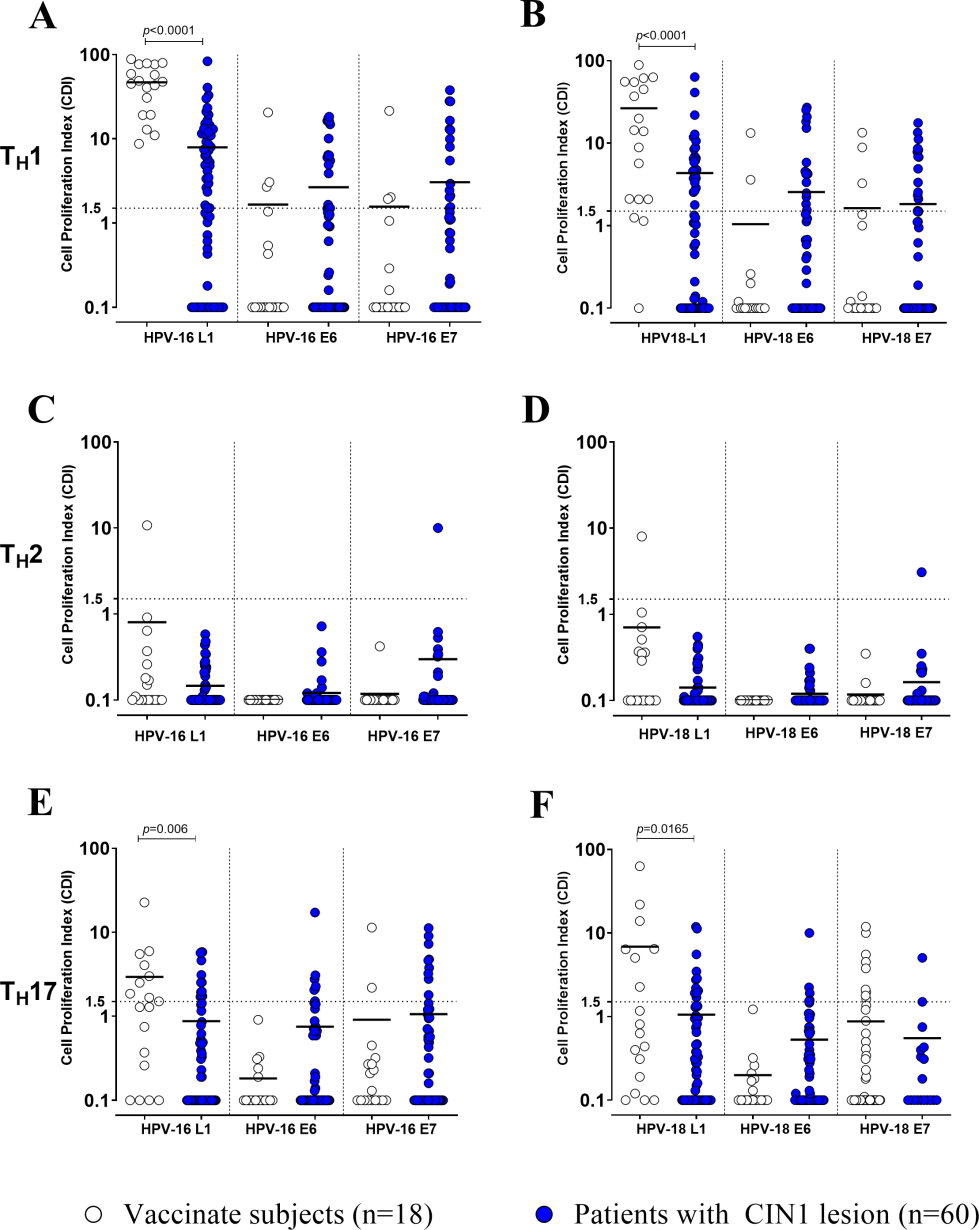
Cell proliferation index (CPI) for TH1 **(A, B)** TH2 **(C, D)** and TH17 **(E, F)** against HPV-16 and - 18 for antigens L1, E6 and E7 was measured using Lymphoproliferation (LPR) in vaccinated women (white dots) and CIN1 patients (blue dots). Significant p value were given for each graph. Dotted horizontal lines indicates cut-off of positive results.

In vaccinated women, CD4^+^ T-cell response against L1 of both HPV-16 and -18 was mainly associated with TH1 and TH17 phenotype, while TH2 was almost undetectable in the large majority of subjects (**Figure 3**). Similarly, CD4^+^ T-cell response against L1 was mainly linked to TH1 and TH17 phenotypes in CIN1 women, with lower levels of response than those detected in vaccinated women (p<0.0001 for TH1, and p=0.006 and p=0.0165 for TH17 against HPV-16 and HPV-18 L1; **Figures 3A, B, E, F**). No difference was observed for HPV-16/18-specific T-cell response against E6 or E7 between vaccinated women and patients with CIN1 lesion when TH1, TH2 and TH17 compartments were compared.

### 3.3 HPV genotype and T-cell response

**Figure 4:**
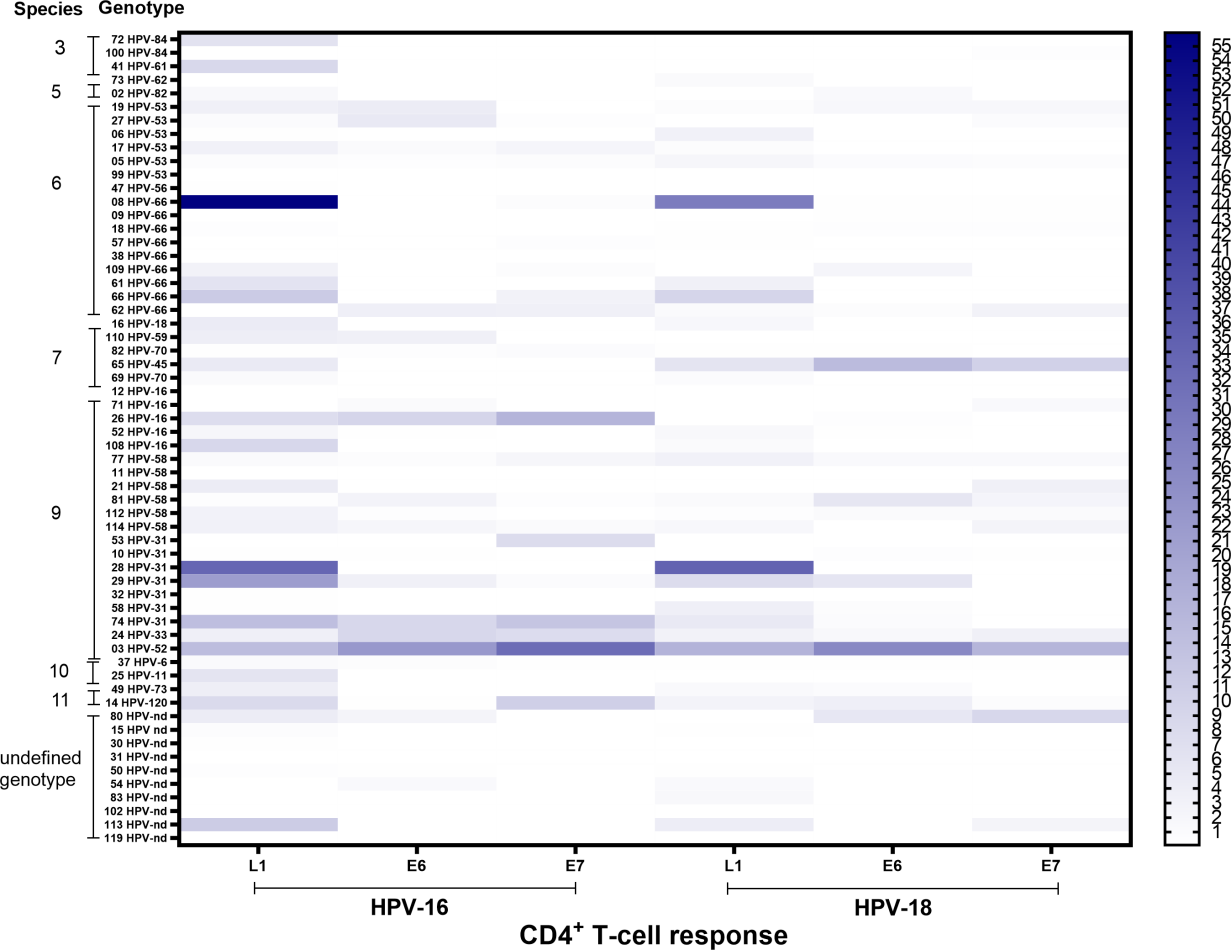
Heat map showing the percentage of CD4^+^ T-cell response against HPV-16 and -18 antigens (L1, E6 and E7) in CIN1 patients according with infecting HPV genotyping. The color scale intensity of the heat map shown on the right side of the graph represents T-cell response, with colors ranging from blue bars (high values) to white bars (low values).

**Figure 5:**
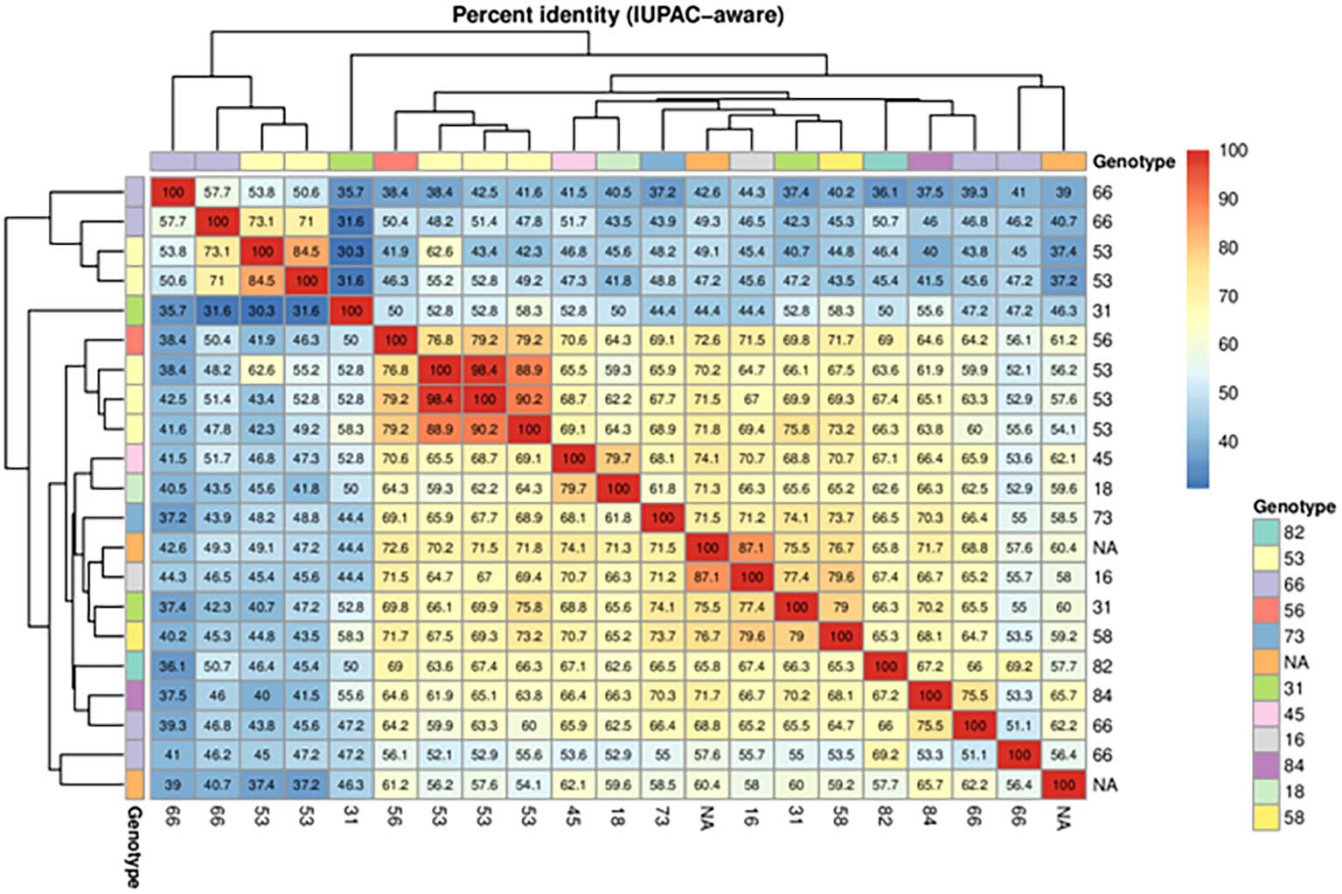
Sequence homology in 19/21 samples using the Sanger method. The sequence homology between different genotypes in the same epitopes was observed.

HPV genotype was defined in 50/60 patients (83.4%) while 10/60 (16.6%) patients showed undefined genotype (**Table 1**). Among the 20 different genotypes detected, HPV-66 (9/60 women, 15%), HPV-31 (7/60, 11.6%), HPV-53 (6/60, 10%), HPV-58 (6/60, 10%), and HPV-16 (5/60, 8.3%) were those most frequently detected. The CD4^+^ T-cell response level and the HPV genotype of individual patients is shown in **Figure 4**. HPV-specific T-cell response was detected also in patients infected by genotypes other than HPV-16 or -18, and levels of T-cell response were independent from the HPV species or genotype responsible for the infection. Moreover, 21 samples were sequenced using the Sanger method, in order to detect the genotype and evaluate the sequence homology between different genotypes in the same epitopes (**Figure 5**). A total of 19/21 (90.4%) sequences belonging to 18 different HPV genotypes (82, 53, 66, 56, 73, 31, 45, 16, 84, 18, 58) were identified. For two samples, it was not possible to derive the genotype (**Figure 5**, NA=not available). Sequence homology was highest between genotypes 53 and 56 (range 76.7%-79.1%), 53 and 31 (75.8%), 31 and 16 (77.3%), 84 and 66 (75.4%), 45 and 18 (79.6%), 31 and 58 (79.0), 16 and 58 (79.6%). One ungenotyped sample showed the highest sequence homology value (87.1%) with genotype 16, while the other sample showed a maximum value of 65.6% with genotype 84.

## 4 Discussion

In this study, we design and implemented a test to evaluate the T-cell response against the L1, E6, and E7 proteins of HPV- types 16 and 18 in vaccinated healthy women and patients with HPV-related CIN1. In all the vaccinated women, a CD4^+^ T-cell response against HPV-16 L1 and, with less extent, against HPV-18 L1 (both included in vaccines formulation) was observed when measured with LPR assay. Of note, a CD8^+^ T-cell response against HPV-16 and -18 L1 was observed in less than 70% of vaccinated women. In addition, even if T-cell response against E6 and E7 antigens was almost undetectable in the large majority of vaccinated women, about 20% of subjects tested positive for this response, suggesting a previous exposure to HPVs. In the cohort of patents with CIN1 here examined, as observed for vaccinated women, L1 antigen was associated with the highest response for both CD4^+^ and CD8^+^ T cells, although it was detected in about half of the subjects. A reduced response was observed instead for E6 and E7 antigens. As expected, median T-cell response after vaccination was significantly higher than that observed after natural infection. Finally, T-cell response to peptides from antigens derived from HPV16 and 18 was detected in CIN1 patients infected by HPV genotypes other than 16 and 18, suggesting a potential T-cell cross reactivity among HPV genotypes.

We firstly compared the performance of AIM assay, designed to detect effector memory-like cells, which are rapidly activated after ex-vivo stimulation, and LPR assay, in which a long-term stimulation of 7 days allows for an expansion of memory T cells. The T-cell response against L1 assessed with the AIM assay was either low or absent, whereas it was detected using LPR assay. This suggests that effector- memory T cells elicited by HPV vaccination do not persist or are maintained at low frequency, below the detection level of the AIM assay. Conversely, HPV-specific T cells with proliferative potential are detectable in most vaccinated subjects, especially within the CD4^+^ T-cell subset. The persistence of virus- specific memory T cells which are detectable with the LPR assay rather than with the AIM assay was already observed in subjects tested some months after SARS-CoV-2 infection (22).

Since HPV-related tumor progression seems to be related to an immune system escape (23), evaluating the T-cell response during the infection phase could be crucial for monitoring potential persistence or progression of the lesion and predict clinical out-come (20, 24–27). Therefore, we decided to investigate HPV-specific T-cell response in a selected cohort of patients with CIN1 lesion, also assessing the HPV type related to the infection. Our results underlined that CD4^+^ T-cell response against L1 antigen was detectedby the LPR assay in about 30 or 50% patients, when peptides from HPV-18 or HPV-16 were used for T- cell stimulation, while CD8^+^ T-cell response was less represented. E6 and E7 proteins appeared less immunogenic, as specific CD4^+^ T-cell response was de-tected only in about 15% patients, and CD8^+^ T cells were not detected.

We performed an analysis considering the group of patients with CIN1, divided by category: menopausal and non-menopausal women, since that these results should be interpreted acknowledging potential confounding by age and immune senescence. However, no statistically significant immunological differences were observed between the two groups.

As reported in several studies, some subsets of CD4^+^ T cells, such as TH1, TH2, and TH17 have important roles in mediating host defensive mechanisms against various infections or in the pathogenesis of various autoimmune diseases (28, 29). Moreover, the imbalance of TH1, TH2 and T_H_17 in cervical cancer patients might be involved in the development and progression of cancer (30–32). We observed that HPV-specific T-cell response against L1, E6 and E7 was mainly associated with the TH1 and, to a lesser extent, with the TH17 phenotype, while TH2 cells were almost negligible in both vaccinated and infected subjects. Whether patients showing TH2 and/or TH17 response to HPV will have an unfavorable outcome need to be further investigated in long-term clinical studies.

The reduced T-cell response observed in patients with respect to vaccinated subjects could be rely on the use of peptides from HPV-16 and HPV-18 only, leading to a potential underestimation of the actual HPV-specific T-cell response in patients infected by other HPV genotypes. However, the level of the proliferative response observed seemed not to be related to the HPV genotype infecting the patient: as an example, the highest responses were observed in patients infected by HPV-66, HPV-31 and HPV-52. Therefore, the T-cell response to peptides from HPV-16 and HPV-18 observed in patients of our cohort, who were infected by several different HPV types, suggests a potential cross-reactivity of HPV-specific T cells and, therefore, a potential cross-protection conferred by investigational HPV therapeutic vaccines. Previous studies reported a cross-reactivity of HPV-16/18 vaccine against other HPV types (33–35). Several studies showed a T-cell cross-reactivity between different HPV types (36, 37). Van den Hende and colleagues investigated the cross-recognition of five closely related members of the species 9 of the alpha genus (HPV-31, -33, -35, -52, and -58). Using overlapping peptides, approximately half of the responding subjects dis-played recognition of more than two other HPV types, suggesting that cross- reactivity may be relatively common. However, cross-reactivity against HPV-16 and HPV-18 oncoproteins E6 and E7 was uncommon in T cells that infiltrate metastatic HPV-16 and -18 positive cancers and in T cells that target the HPV oncoproteins, supporting a change in immunotherapy clinical trial practice to match the HPV type of the oncoprotein vaccine with that of the tumor (38). Mechanisms of cross-protection conferred by investigational HPV therapeutic vaccines was debated. The cross- reactivity of T cells induced by HPV therapeutic vaccines was expected to target epitopes of HPV types with high amino acid sequence homology (36).

Another observation that arises from our study is that HPV-16 and HPV-18 are poorly represented among patients with CIN1 lesions, while other genotypes appear to play a major role, as reported in other studies (39, 40). The reduced prevalence of HPV-16 in CIN1 lesions and the corresponding increase of the prevalence of HPV types unrelated to nonavalent vaccine and of untypable HPV was recently described in Northern Italy, suggesting a potential replacement of HPV-16 by other HPV (41). This modification was possibly ascribed to demographic factors and vaccination. The results presented in this study have some limitations, such as the low number of CIN1 patients and vaccinated subjects examined, the lack of follow-up the lack of sequence analysis. Furthermore, the B-cell response was not investigated. Nevertheless, the LPR assay here described could be adopted to evaluate the HPV-specific immune response in patients with CIN1 in order to define the potential association between lesion resolution or progression and T-cell response, and identification of novel prognostic parameters. Moreover, investigating sequence homology between HPV-16 and HPV-18 with the HPV types detected in lesions, and the identification of conserved epitopes, will be important to assess the potential for T-cell cross- reactivity, particularly in the context of a therapeutic vaccination (42–44).

In conclusion, in this study we have design and optimized a test to evaluate the T-cell response against the L1, E6, and E7 proteins of HPV types 16 and 18 in vaccinated healthy women and patients with CIN1 lesions. Using the LPR test, all vaccinated sub-jects showed a stronger response compared to the patients. Moreover, we observed a T-cell response to peptides from HPV-16 and HPV-18 in patients despite being infected by several different HPV types. Although the study requires sequence analysis of the HPV epitopes, these results suggest a potential cross-reactivity of HPV-specific T cells. Thus, these findings may reflect a potential cross-protection offered by investigational HPV therapeutic vaccines.

## Data Availability

The raw data supporting the conclusions of this article will be made available by the authors, without undue reservation.

## Supplementary material

Supplementary Figure 1 Cell proliferation index (CPI) for both CD4^+^**(A, B)** and CD8^+^ T cells **(C, D)** against HPV-16 and -18 for antigens L1, E6 and E7 was measured using Lymphoproliferation (LPR) in no menopausal women (dark gray dots) and menopausal women (light gray). Significant p value were given for each graph. Dotted horizontal lines indicates cut-off of positive results.

Supplementary Figure 2 The flow cytometry analyses and gating strategy using Lymphoproliferation was observed. The frequency of CD4^+^ICOS^+^CD25^+^ and CD8^+^ICOS^+^CD25^+^ was reported.
