# Supplementary figures and images for: Detection of human papillomavirus (HPV)-specific T cell response in women with low-grade cervical intraepithelial lesion and HPV vaccinated subjects
Source: Front Immunol. 2026 Feb 12;16:1733404. doi: 10.3389/fimmu.2025.1733404 (PMC12935884; doi:10.3389/fimmu.2025.1733404)

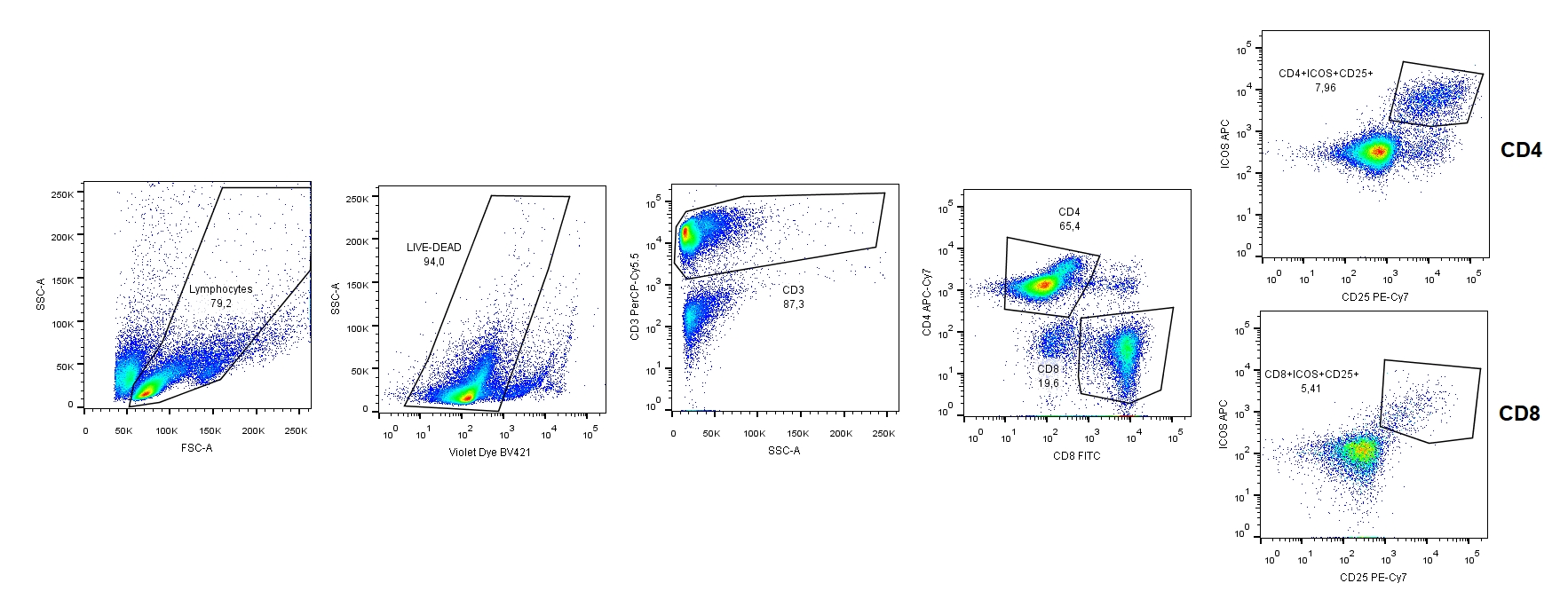

Supplement: Supplementary Figure 1 — Cell proliferation index (CPI) for both CD4+(A, B) and CD8+ T cells (C, D) against HPV-16 and -18 for antigens L1, E6 and E7 was measured using Lymphoproliferation (LPR) in no menopausal women (dark gray dots) and menopausal women (light gray). Significant p value were given for each graph. Dotted horizontal lines indicates cut-off of positive results. [file Image1.tif]

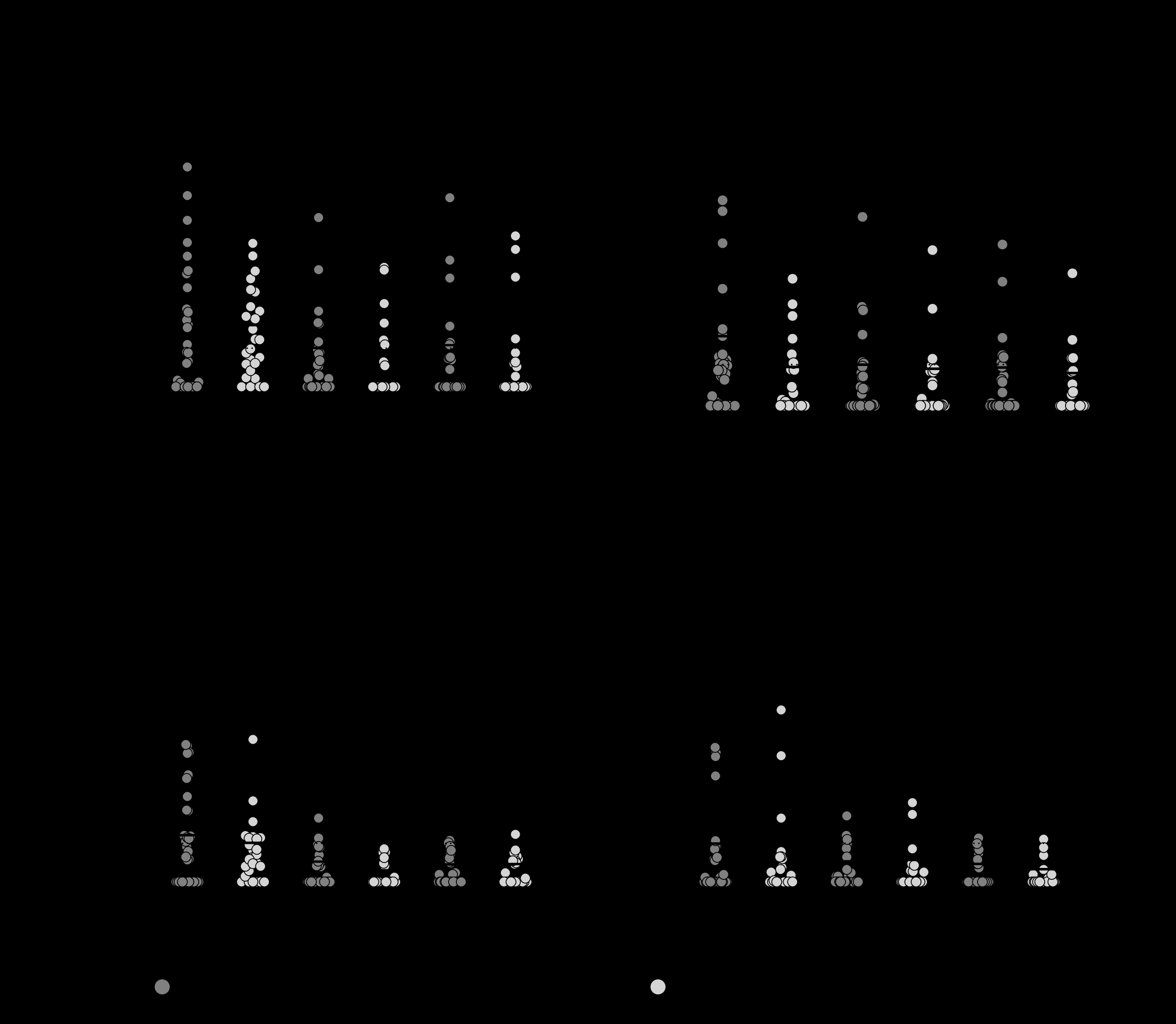

Supplement: Supplementary Figure 2 — The flow cytometry analyses and gating strategy using Lymphoproliferation was observed. The frequency of CD4+ICOS+CD25+ and CD8+ICOS+CD25+ was reported. [file Image2.tif]
